# Antibacterial and Photodegradation of Organic Dyes Using Lamiaceae-Mediated ZnO Nanoparticles: A Review

**DOI:** 10.3390/nano12244469

**Published:** 2022-12-16

**Authors:** Dorcas Mutukwa, Raymond T. Taziwa, Lindiwe Khotseng

**Affiliations:** 1 Department of Chemistry, University of the Western Cape, Robert Sobukwe Rd., Private Bag X17, Bellville 7535, South Africa; 2 Department of Applied Science, Faculty of Science Engineering and Technology, Walter Sisulu University, Old King William Town Road, Potsdam Site, East London 5200, South Africa

**Keywords:** plant-mediated synthesis, nanoparticles, ZnO nanoparticles, Lamiaceae family, photodegradation, antibacterial, green synthesis, biosynthesis, biomolecules, phytochemicals, organic dyes, photocatalyst

## Abstract

The green synthesis of zinc oxide nanoparticles (ZnO NPs) using plant extracts has been receiving tremendous attention as an alternative to conventional physical and chemical methods. The Lamiaceae plant family is one of the largest herbal families in the world and is famous for its aromatic and polyphenolic biomolecules that can be utilised as reducing and stabilising agents during the synthesis of ZnO NPs. This review will go over the synthesis and how synthesis parameters affect the Lamiaceae-derived ZnO NPs. The Lamiaceae-mediated ZnO NPs have been utilised in a variety of applications, including photocatalysis, antimicrobial, anticancer, antioxidant, solar cells, and so on. Owing to their optical properties, ZnO NPs have emerged as potential catalysts for the photodegradation of organic dyes from wastewater. Furthermore, the low toxicity, biocompatibility, and antibacterial activity of ZnO against various bacteria have led to the application of ZnO NPs as antibacterial agents. Thus, this review will focus on the application of Lamiaceae-mediated ZnO NPs for the photodegradation of organic dyes and antibacterial applications.

## 1. Introduction

Nanomaterials (NMs) have been receiving tremendous attention globally in research owing to the quantum confinement effect, which predominates at the nanoscale and gives rise to unique magnetic, structural, and optical properties of these materials [1]. Furthermore, the dominance of size confinement, morphology, distribution, and interfacial effects at the nanoscale also results in unique properties of NMs when compared to their bulk counterparts. Another characteristic that makes NMs intriguing is the ability to control and manipulate the size, morphology and distribution, and interfacial effects, which yield a variety of physical, chemical, and biological properties [2]. Thus, NMs have found applications in medicine [3,4], environment [5,6], energy [7,8], agriculture [9,10], textile [11,12], gas sensors [13,14], and food [15,16] industries.

ZnO nanoparticles (NPs) have grown in prominence owing to their low toxicity and environmental friendliness. The multi-versatility of ZnO NPs has resulted in their applications in a wide variety of industries and disciplines. ZnO NPs have managed to replace their bulk counterpart in a wide range of applications owing to their small size, which results in a high surface-to-volume ratio, leading to increased efficiency [17]. ZnO NPs have found use as gas sensors due to their high electron mobility, good chemical stability, good electrical properties, and good thermal stability [18,19]. The large bandgap of ZnO (3.37 eV) and high binding energy (60 meV) result in its application in optoelectronic devices [20], such as biosensors and bioimaging [21]. Due to its strong luminescence in the visible region of the light spectrum, it can be used as a phosphor, and it can also be employed in frequency converters owing to its non-linear optical response. It is also used as an additive in rubber owing to its high thermal conductivity [22]. The transparency properties of ZnO and good ultraviolet (UV) radiation absorption have resulted in its use as an UV protector in cosmetics and textiles [23]. The high surface reactivity of ZnO and the ability to create electron-hole pairs make it an excellent candidate for photocatalysis [24]. Additionally, ZnO NPs possess the potential for medical application owing to their antibacterial, anticancer, antioxidant, and antidiabetic properties [25].

A wide range of synthesis methods for ZnO NPs have been documented in the literature, and these include physical, chemical, and biological methods. However, recent research has been focused on green synthesis methods that eliminate the use of hazardous chemicals and reduce energy consumption. This is due to the fact that conventional synthesis methods utilise hazardous chemicals and require high energy input [26]. Biological synthesis is a green synthesis approach that utilises plants, bacteria, fungi, or algae for the preparation of NPs and is a safer and cheaper option than the conventional physical and chemical methods [27].

Plants such as *Laurus nobilis* [28], *Tecoma castanifolia* [29], *Cassia fistula* [30], *Deverra tortuosa* [31], *Azadirachta indica* [32], *Aspalathus linearis* [33], *Moringa oleifera* [34], Euphorbia hirta [35], Aloe vera, and Hibiscus sabdariffa [36] have successfully been used for the preparation of ZnO NPs. The plants belong to various families such as Lauraceae, Bignoniaceae, Leguminosae, Apiaceae, Meliaceae, Fabaceae, and Moringaceae, etc. The plants are capable of synthesising ZnO NPs owing to the presence of secondary metabolites, which can be used as reducing or stabilising agents during ZnO NPs preparation [17]. The Lamiaceae family (Labiate), generally known as the mint family, is famous for its medicinal aromatic plants that are spread throughout the world [37,38]. Some of the popular Lamiaceae plants include herbs such as *Ocimum basilicum* (basil), *Salvia officinalis* (sage), *Origanum vulgare* (oregano), *Mentha* (mint), and *Rosmarinus officinalis* (rosemary) [39]. The Lamiaceae plants have been used for the plant-mediated synthesis of ZnO NPs in addition to their use in traditional and modern medicine, food, and pharmaceutical industries [40]. Thus, this review will discuss the Lamiaceae-mediated ZnO NPs.

Organic dyes are a major pollutant found in wastewater from the textile, rubber, cosmetics, leather, pharmaceutical, and printing industries [41]. More than 10–15% of the global 7 × 10^5^ tonnes of organic dyes produced annually end up as waste. These organic dyes make their way into the environment, where they cause adverse harm to humans, flora, and fauna [42]. The removal of these harmful substances from wastewater is very crucial in order to safeguard life and the environment. However, the conventional methods for removal of organic dyes, such as adsorption, ion flotation, sedimentation, coagulation, flocculation, etc., fail to fully degrade the organic dyes or result in the production of high volumes of sludge, and some of these methods are cost ineffective [43,44,45]. As a result, the photodegradation of organic dyes using photocatalysts has been getting attention since it results in the complete degradation of the organic dyes and is cost-effective and environmentally friendly. Properties of ZnO, such as a wide direct bandgap (3.37 eV), high quantum efficiency, unique electrical, optoelectronic and luminescence properties, and environmental friendliness make ZnO an excellent candidate for photodegradation of organic dyes [45,46].

Additionally, because of the biocompatibility, high thermal stability, high selectivity, low cytotoxicity, and biodegradable nature of ZnO NPs, the NPs have also found use as antibacterial agents [47,48]. There has been a global surge in antibacterial-resistant microorganisms [49], necessitating the development of novel antibacterial agents to protect human health. Many studies on the antibacterial activity of plant-mediated ZnO NPs have been published, and these include *Zingiber officinale* [50], *Carissa spinarum* [51], *Pongamia pinnata* [52], *Azadirachta indica* [32], and *Lepidium sativum* [53]. This review will look at the extraction process of biomolecules from Lamiaceae plants as this process can influence the synthesis of ZnO NPs. The synthesis of ZnO NPs using plants belonging to the Lamiaceae family will be discussed, as will the influence of reaction parameters on the size, morphology, and properties of the Lamiaceae-mediated ZnO NPs. This is because reaction parameters such as pH, precursor concentration, reaction temperature, calcination temperature, plant extract concentration, etc., can be controlled to produce high-quality NPs and NPs suitable for their intended applications [54,55]. The review will also include the common structural, morphological, and optical characterisation techniques used for the characterisation of Lamiaceae-mediated ZnO NPs. Moreover, applications of the Lamiaceae-mediated ZnO NPs in the photodegradation of organic dyes and antibacterial will be discussed.

## 2. Synthesis of Methods of ZnO NPs

### 2.1. Physical Methods

Generally, physical synthesis methods can be categorised under top-down and bottom-up synthesis approaches. The top-down approach entails the breaking down of bulk matter into smaller particles until the nanosize is achieved. The bottom-up synthesis approach involves the build-up of nanosized particles from ions, molecules, and atoms via nucleation. Laser ablation, ball milling, lithography, mechanical grinding, and ion beam technique are some of the physical synthesis methods that have been employed for the preparation of ZnO NPs [56]. The laser ablation method entails focusing a laser beam on a high-purity zinc plate that is immersed in a liquid under a vacuum. This leads to the formation of a plasma, which creates species that can react with the liquid, resulting in the formation of NPs [57]. Wirunchit et al. [58] synthesised spherical ZnO NPs with a crystallite size of 68.4 nm using zinc (Zn) metal as a precursor and zirconia balls for crushing. The Zn metal precursor was crushed for 18 h in the presence of ethylene alcohol lubricant before being calcined at 800 °C in oxygen to form the ZnO NPs. Most of the physical synthesis methods do not involve hazardous solvents but they are hampered by high energy consumption and extended periods of time necessary to perform NPs synthesis.

### 2.2. Chemical Methods

Most chemical synthesis methods for the preparation of ZnO NPs fall under the bottom-up approach. The methods include chemical reduction, hydrothermal, sol-gel, sonochemical, microwave-assisted, etc. The sol-gel method is a low-temperature and easy method that involves the polycondensation of a hydrolysed organometallic precursor. The hydrolysis process can be achieved using water or alcohol in the presence of a base or acid. The polycondensation process results in the formation of a gel, and the NPs formation can be accomplished through the ageing, solvent extraction, and drying steps [47]. Using the sol-gel method, Al Abdullah et al. [59] synthesised ZnO NPs with 12–30 nm average particle size. The method involved mixing a methanol solution with zinc acetate dihydrate (Zn(CH_3_COO)_2_·2H_2_O) and stirring at 80 °C for 2 h to create a sol. The pH was adjusted between 9 and 11 using ammonia (NH_4_OH), and the sol was dried at 100 °C for 1 h. The temperature was then raised to 150 °C to obtain the gel, which was subsequently evaporated to produce the white precipitate. To obtain the ZnO NPs, the precipitate was dried at 200 °C and subsequently annealed at 500 °C. Another chemical method that has been used for the preparation of ZnO NPs is the sonochemical method. It is a safe and rapid method that utilises ultrasonic irradiation to produce cavities in a liquid medium. The NPs can be produced in the following three steps: cavity generation, growth, and collapse. When the liquid is irradiated with ultrasonic waves, there is a massive energy increase to about 5000 K temperature and 20 MPa pressure, which will end up collapsing the cavities and resulting in chemical excitation and NPs formation [60]. Noman et al. [61] synthesised uniform quasi-spherical ZnO NPs with an average particle size of 28 nm using a one-pot ultrasonic homogeniser at 20 kHZ, 200 W, and 50% efficiency with zinc chloride (ZnCl_2_) and sodium hydroxide (NaOH).

Chemical reduction is a widely used chemical synthesis method for the preparation of ZnO NPs. The method employs reducing agents such as hydrazine, sodium borohydride, Tollen’s reagent, N,N-dimethylformamide, and sodium citrate and stabilising/capping agents such as polyethylene glycol, polyvinyl alcohol, and polyvinyl pyrrolidone [62]. The reducing agents reduce the Zn salt precursors, and the purpose of the stabilising agents is for the stabilisation of the synthesised NPs to prevent agglomeration and steric hindrance. Mornami et al. [63] synthesised ZnO NPs by reducing zinc nitrate hexahydrate (Zn(NO_3_)_2_·6H_2_O) with hydrazine and stabilising the synthesised NPs using ethylene glycol. The synthesised ZnO NPs had particle size between 46 and 66 nm. Chemical synthesis methods are widely studied and utilised for the synthesis of ZnO NPs because of their advantages, which include the easy control of particle size and morphology of nanoparticles. However, some of the chemical synthesis methods make use of high temperatures and pressures, toxic reducing and stabilising agents, which are harmful to humans and the environment. The use of toxic stabilising agents can result in some toxic chemicals being adsorbed on the surface of the NPs, limiting their use for medical applications [64]. As a result, current efforts have concentrated on the development of innovative synthesis methods to address the drawbacks of physical and chemical synthesis methods.

### 2.3. Biological Methods

Biological synthesis methods utilise natural products such as plants, algae, and microbes. The process makes use of functional groups such as amino, hydroxyl, amine, and carboxylic from biomolecules derived from the natural products to reduce Zn salt precursors and stabilisation of the NPs. As noted below, this approach offers significant benefits over traditional physical and chemical synthesis methods as follows [54]:

- Low cost;
- Environmentally friendly;
- Easily scalability;
- Low energy consumption;
- Easy availability of resources;
- Simple and rapid.

The preparation of ZnO NPs using microbes has been reported and some of the microbes reported include bacteria; *Bacillus megaterium* [65], *Lactobacillus plantarum* VITES07 [66], *Serratia nematodiphila* [67], yeast; *Pichia kudriavzevii*, *Pichia fermentans* JA2 [68], fungi; *Acremonium potronii* [46], *Periconium sp* [69]. Biosynthesis of ZnO NPs using bacteria can be achieved via intracellular and extracellular synthesis. The intracellular bacteria synthesis involves the bacteria cell wall and ion transporters. The intracellular bacteria method is limited, owing to the additional processing necessary to obtain the ZnO NPs from the bacteria, which results in a longer synthesis time and a higher cost. Extracellular bacteria synthesis, on the other hand, occurs outside the bacteria and involves the use of enzymes on the cell membrane or enzymes released by the bacteria into the growth medium. While this technique is quick and easy, it is complicated by the possibility of NP contamination by compounds from the bacteria in the growth medium [70].

Plants parts such as leaves, roots, seeds, peels, fruits, stems, and flowers are known to contain phytochemicals such as flavonoids, phenols, alkaloids, proteins, terpenoids, and saponins that can be extracted and used to reduce Zn salt precursors and stabilise the NPs. Plant-mediated synthesis, in contrast to microbe-mediated synthesis, is simple and does not require additional complicated processes, such as culture growth and maintenance. Plant-mediated NPs have been reported to exhibit better antibacterial activity and catalytic activity than chemically synthesised NPs [71,72]. Hence, this review will focus on the plant-mediated synthesis of ZnO NPs using plants from the Lamiaceae family, as well as their applications for antibacterial activity and photodegradation of organic dyes.

## 3. Lamiaceae-Mediated Synthesis of ZnO NPs

### 3.1. Preparation of Plant Extracts

In general, phytochemicals from plants can be extracted using a variety of solvents such as water, acetone, methanol, ethanol, ether, and petroleum. When it comes to the solvent extraction of phytochemicals, “like dissolves like”, which means that polar solvents target polar molecules, while non-polar solvents target non-polar compounds. Alcohol-based extraction with ethanol yields phytochemicals such as saponins, tannins, phenones, polyphenols, terpenoids, etc. [73]. Solvent selection is an important aspect of plant-mediated synthesis of NPs, and this is because it affects the composition and concentration of the phytochemicals involved in the reduction and stabilisation of NPs during synthesis [74]. Water is the most common solvent used for the extraction of the phytochemicals from the Lamiaceae family of plants. This is not unexpected given that water is the most often used solvent for phytochemical extraction in plant-mediated NP synthesis. This is because water is non-toxic, non-flammable, and cost-effective. Moreover, because water is a polar solvent, it can yield biomolecules such as polyphenols, which are capable of reducing Zn salt precursors during synthesis.

El-Gammal [75] reported the extraction of phytochemicals from *R. officinalis* using ethanol and water as solvents. Even though water is more polar than ethanol, the ethanol extracts of *R. officinalis* had a higher concentration of both phenolic compounds, such as caffeic acid, vanillic acid, cinnamic acid, and catechol, as well as flavonoids such as rosmaric, quercetin, rutin, and hesperetin. However, the obtained ZnO NPs prepared using ethanoic and water extracts of *R. officinalis* did not exhibit any significant differences, with particle sizes ranging from 2.8 to 3.8 nm according to the transform electron microscopy (TEM) results. A similar observation was reported for the *Severinia buxifolia* plant, which belongs to the Rutaceae family, where the methanol extracts exhibited a higher concentration of phytochemicals as compared to the water extracts [76]. Methanol has also been used for the extraction of phytochemicals from *Mentha viridis,* which belongs to the Lamiaceae family [77]. However, the authors did not report on the phytochemical compounds present in the methanoic extracts. Hou et al. [78] extracted proteins from *Perilla frutescens* for ZnO NPs synthesis using petroleum ether. The choice of solvent for the extraction of biomolecules from plants is dependent on the targeted biomolecules.

The extraction conditions depend on the desired biomolecules. Temperature is one crucial parameter that affects the yield of biomolecules in the plant extract. This is because an increase in temperature increases phytochemical diffusion and solubility in the extraction solvent. However, extremely high temperatures can result in damage to some heat-sensitive phytochemicals [74]. Therefore, an optimum temperature is necessary for the extraction of phytochemicals from plants. The majority of the phytochemical extractions from Lamiaceae plants were performed at temperatures ranging from room temperature (RT) to 100 °C. However, there were no reports of optimisation of the extraction parameters. There is a need for an optimisation of the extraction parameters such as extraction time, temperature, and extraction solvent of the Lamiaceae plants, and this is particularly important because it eliminates needless heating and contact time, making the process more cost-effective while assuring high-quality yield [79].

### 3.2. Synthesis of Lamiaceae-Mediated ZnO NPs

Generally, most of the Lamiaceae-mediated ZnO NPs were prepared by heating a mixture of the plant extracts and the Zn salt precursors to obtain a precipitate. The ZnO NPs can be obtained by thermal decomposition and annealing the precipitate in a furnace. The heating source can be a hotplate, an autoclave, or a microwave. Mohammadi-Aloucheh et al. [80] synthesised ZnO NPs using the *Mentha longifolia* extract using a microwave-assisted method. The method involved dissolving 7.31 g of Zn(NO_3_)_2_·6H_2_O Zn salt precursor in a solution of 20 mL *M. longifolia* extract and 80 mL distilled water and adjusting the pH to 10 using a 5 M NaOH solution. The mixture was irradiated in a microwave at 2.45 GHz and 1000 W. A light brown precipitate was obtained and centrifuged at 5000 rounds per minute (rpm) for 8 min. This was followed by cycles of washing the precipitate with ethanol and distilled water and finally drying it in the oven at 60 °C for 24 h. In another study, Stoyanova and associates [81] reported hydrothermally synthesised *Mentha arvensis*-mediated ZnO NPs. The method involved adding a solution of 0.2 M Zn(CH_3_COO)_2_·2H_2_O and 0.6 M urea at pH 8 to varied extract concentrations of *M. arvensis* and stirring at 200 rpm. The obtained mixtures were autoclaved at 180 °C and the obtained precipitates were washed with distilled water. The washed precipitates were then oven-dried and finally calcined at 170 °C for 3 h. Tang et al. [82] synthesised ZnO NPs using *Scutellaria baicalensis* plant extracts. The method involved adding 100 mL of 0.1 M Zn(CH_3_COO)_2_·2H_2_O to 20 mL of *S. baicalensis* extract and maintaining the mixture at a pH of 11 with continuous stirring at 80 °C for 3 h. Some of the Lamiaceae-mediated synthesis of ZnO NPs can even be performed at RT, as demonstrated by Lučić et al. [26], who synthesised ZnO NPs at RT using 115 mL of Zn(CH_3_COO)_2_·2H_2_O and 50 mL of *Ocimum basilicum* extract. There was no change in colour; however, a white precipitate of ZnO NPs was observed. The majority of the Lamiaceae-mediated synthesis was performed at temperatures ranging between 60 and 100 °C and calcined at temperatures from 200 to 500 °C. This demonstrates that ZnO NPs can be effectively synthesised by utilising plants without the need for hazardous chemicals or high temperatures.

### 3.3. Factors Affecting Synthesis of Lamiaceae-Mediated ZnO NPs

This section will discuss the factors, which affected Lamiaceae-mediated ZnO NPs. The Lamiaceae-mediated ZnO NPs similar to other plant-mediated ZnO NPs, were affected by factors such as pH, plant extract concentration, precursor concentration, reaction temperature, and calcination temperature.

**Figure 1:**
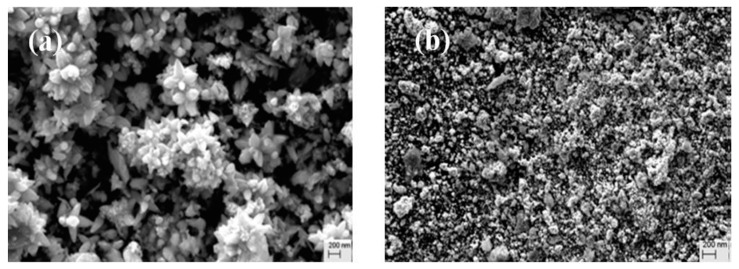
Scanning electron microscopy (SEM) images of (**a**) chemically derived ZnO NPs and (**b**) *O. tenuiflorum*-mediated ZnO NPs. Adapted from [84].

Zheng et al. [83] investigated the plant-mediated synthesised and the chemically synthesised ZnO NPs. The different synthesis routes resulted in ZnO NPs of different morphologies, with the *Plectranthus amboinicus*-mediated ZnO NPs being rod-shaped with an average particle size of 88 nm whereas the chemically synthesised ZnO NPs were spherical with an average particle size of 74 nm. Comparative studies between the plant-mediated and the chemical synthesis were also reported by Upadhaya et al. [84]. The plant-mediated synthesis was performed using *Ocimum tenuiflorum* leaf extracts. The *O. tenuiflorum* extract was boiled at 60–70 °C and 5 mg of zinc nitrate was added at 70 °C. A deep reddish paste was obtained and dried at 100–130 °C for 45 min to obtain yellowish ZnO NPs. The chemical synthesis was performed by heating a mixture of thiol-urea, Zn(CH_3_COO)_2_·2H_2_O and NH_4_OH in a 1:1:1 ratio to 80 °C. The ZnO NPs were obtained after washing with acetone, ethanol, and deionised water. The chemically synthesised ZnO NPs had larger crystallite sizes compared to the *O. tenuiflorum*-mediated ZnO NPs. The differences in morphologies were also noted, with the chemically derived ZnO NPs exhibiting nanoflowers with some agglomeration and the *O. tenuiflorum*-mediated ZnO being spherical and agglomerated, as shown in Figure 1.

In other studies, a comparative investigation of the crystallite size and morphology of ZnO NPs synthesised with and without *Mentha spicata* extracts was performed by Karaköse and Çolak [85]. The *M. spicata*-mediated ZnO NPs were rod shaped with a smaller average crystallite size of 50 nm, whereas the ZnO NPs synthesised without the plant extract were granular and circular with some aggregates and a larger average crystallite size of 60 nm. These findings demonstrate that plant extracts can successfully reduce and stabilise ZnO NPs.

The green synthesis of ZnO NPs using Zn salt precursors such as zinc acetate, zinc nitrate hexahydrate, zinc chloride, and zinc sulphate has been studied widely, as shown in Table 1. According to Saba et al. [86], there are no significant differences in the crystallite size of the ZnO NPs obtained using different Zn salt precursors, while different Zn salt precursors may result in a significant influence on the morphology of the obtained ZnO NPs. This is in agreement with findings reported by Sekar et al. [87] for *Anisomeles malabaria*-mediated ZnO NPs. Using Zn(CH_3_COO)_2_ precursor resulted in small spherical particles that grew into bullets, whereas using Zn(NO_3_)_2_ resulted in round particles that accumulated into flowers.

The pH is an important parameter during synthesis as it influences the properties of ZnO NPs. The Lamiaceae-mediated synthesis of ZnO NPs was predominantly performed at an alkaline pH. This is due to several reports that suggest ZnO NPs synthesis favour alkaline conditions. This might be because at acidic pH there are insufficient OH^−^ ions to attract Zn^2+^ and promote the Zn–O bond structure formation, whereas, at alkaline pH, there are sufficient OH^−^ ions [88]. This is supported by findings by Sushma et al. [89], who studied the effect of pH on the optical properties of *O. tenuiflorum*-mediated ZnO NPs. No characteristic peaks of ZnO NPs were observed from the Ultraviolet-visible spectroscopy (UV-Vis) spectra at acidic pH, between pH 4 and 6, indicating that ZnO NPs were not formed. However, peaks were obtained at alkaline pH, from pH 8 to 12, from 360 to 380 nm and it was observed that there was a blue-shift as the pH increased with a sharp peak observed at pH 12. Siddiquah et al. [90] synthesised ZnO NPs at a pH of 12 using in vitro-derived *Isodon rugosus* plant extracts and Zn(CH_3_COO)_2_·2H_2_O. According to the authors, a higher pH favoured a larger number of functional groups from the plant extract biomolecules, which are responsible for the stabilisation of the ZnO NPs, leading to greater stability. However, the mechanism of plant-mediated synthesis is not fully understood yet; therefore, it is difficult to deduce how this happens. In another study, ZnO NPs were synthesised using NaOH, Zn(CH_3_COO)_2_·2H_2_O as precursor solutions, and *Ocimum gratissimum* leaf extracts. The reactions were carried out at varying pH of 8, 10, and 12, precipitates were obtained, maintained at 50 °C for 9 h and ultimately dried in the oven for 7 h. The authors observed an increase in absorbance with an increase in pH from the UV-Vis analysis whereas, particle size from the TEM analysis decreased with an increase in pH [91].

To the best of our knowledge, most of the studies using Lamiaceae plant extracts for the synthesis of the ZnO NPs did not report on the effect of Zn salt precursor concentration on the obtained ZnO NPs. However, Sharma et al. [92] investigated the effect of the Zn salt precursor concentration on the ZnO NPs synthesised using *O. tenuiflorum* and Zn(CH_3_COO)_2_·2H_2_O as the Zn salt precursor. The authors observed that there were no significant differences in the crystalline structure for the varying Zn salt precursor concentrations of 5, 10, and 50 mmol kg^−1^. Nonetheless, there were significant differences in the morphology of the *O. tenuiflorum*-mediated *Z*nO NPs at different Zn salt precursor concentrations. According to the SEM results, *O. tenuiflorum*-mediated ZnO NPs synthesised with 5 mmol kg^−1^ Zn salt precursor were nanospheres and nanomushrooms of varying dimensions together with a few clusters of tiny nanoparticles. While the ZnO NPs synthesised using 10 and 50 mmol kg^−1^ Zn salt precursors were rough with spherical particles of ZnO NPs that agglomerated to a sponge-like morphology and nanocapsules that aggregated to form voids, respectively [92].

The reaction temperature is another parameter that can affect the size, morphology, and properties of ZnO NPs. Few studies of Lamiaceae-mediated synthesis of ZnO NPs have been reported on the effect of reaction temperature on the structure, morphology, and properties of ZnO NPs. Algarni et al. [93] reported *R. officinalis*-mediated ZnO synthesised by heating at 80 °C and *R. officinalis*-mediated ZnO synthesised by autoclaving at 180 °C. According to the TEM results, the ZnO NPs prepared at 80 °C were observed to be predominantly spherical with particle sizes ranging from 18 to 40 nm whereas ZnO NPs prepared at 180 °C were mostly elongated with particle sizes from 20 to 123 nm. The particle size of the ZnO NPs increased with an increase in reaction temperature. NPs with irregular morphology and agglomerated spherical particles were observed from the SEM images for ZnO NPs synthesised at 80 °C, whereas flaky and agglomerated NPs were observed from the SEM images for ZnO synthesised at 180 °C. However, Mtavangu and associates [94] observed a decrease in particle size as reaction temperature increased for *Tetradenia riperia* leaf extract-mediated ZnO NPs. The ZnO NPs were synthesised using Zn(NO_3_)_2_·6H_2_O and *T. riperia* leaf extract at pH 5.10. The mixture was adjusted to pH 7–8 and stirred at different temperatures of RT (30 °C), 60 °C, and 80 °C for 30 min, followed by ageing for 24 h. The precipitate was collected via centrifugation and then dried and annealed in a furnace. The differences in observation could be attributed to the very high temperature employed for *R. officinalis*-mediated ZnO NPs at 180 °C, at higher temperatures agglomeration of the nuclei may occur as a result of faster collisions due to the increased surface activity of the nuclei, thus resulting in larger particle sizes.

The influence of plant extract concentration was investigated by Ramana et al. [95], who synthesised ZnO NPs using *O. gratissimum* leaf extracts. The ZnO NPs were synthesised by mixing aqueous solutions of 10 M NaOH and 0.5 M ZnCl_2_ and the resultant solution was added to different leaf extract concentrations with the extract to distilled water ratio of 0, 1:4, 1:16, 1:30, 1:50, and 1:70. The different leaf extract concentrations resulted in different morphologies, such as nanorods, nanoflowers, prismatic, pyramids, and prisms. The influence of plant extract concentration on the morphology and size of ZnO NPs was also demonstrated in a study by Luque et al. [96], in which a decrease in particle size with an increase in plant extract concentration was observed for *O. vulgare* leaf extract-mediated ZnO NPs. The ZnO NPs were synthesised using 0.1%, 0.5%, and 4% *O. vulgare* leaf extract and Zn(NO_3_)_2_·6H_2_O solution in a water bath at 60 °C until a paste was visible. This was followed by calcination of the paste at 400 °C for 1 h. The plant extract concentration also had an influence on the optical properties of the *O. vulgare*-mediated ZnO NPs. The calculated bandgap from the UV-Vis analysis increased with an increase in plant extract concentration. Nava et al. [97] reported the influence of plant extract concentration on the bandgap and particle size of ZnO NPs. The ZnO NPs were prepared using 0.5, 1, and 2% *M. spicata* plant extracts and Zn(NO_3_)_2_·6H_2_O in a 1:1 ratio. The solutions were stirred for an hour, dried in a thermal bath at 60 °C for 12 h, and finally calcined at 400 °C for 1 h. The particle sizes increased with an increase in the plant concentration, as indicated by the diameters from the TEM results of 20.3, 54.2, and 67.7 nm for 0.5, 1, and 2% *M. spicata* plant extracts, respectively. This resulted in a decrease in bandgap from 3.71 to 3.43 eV as the plant concentration increased. This was attributed to the larger diameter, the smaller the bandgap owing to the effect of surface resonance. Anbuvannan and co-workers [98] synthesised ZnO NPs using 30, 40, and 50 mL of *Anisochilus carnosus* leaf extracts and 5 g Zn(NO_3_)_2_·6H_2_O. There was a decrease in crystallite size (56.14, 49.55, and 38.59 nm for 30, 40, 50 mL plant extract, respectively) with an increase in plant extract according to the X-ray diffraction (XRD) data.

Calcination temperature is also known to affect the size, morphology, and properties of ZnO NPs. Karam and Abdulrahman [99] synthesised ZnO NPs using *Thymus vulgaris* and calcined the ZnO NPs at 150, 250, 350, and 450 °C and observed the change in morphologies. According to the field emission scanning electron microscopy (FESEM) and the XRD, the average particle size increased from 39.4 to 51.86 nm and the average crystallite size increased from 35.2 to 43.3 nm, respectively, as the calcination temperature increased. The bandgap decreased from 2.7 to 2.6 eV as the calcination temperature, which is, however, a small reduction. The ZnO NPs calcined at 450 °C were spherical, uniform in shape and size and exhibited a better quality compared to the low-temperature calcined ZnO NPs. In another study, Kamarajan and co-workers [100] studied the effect of calcination temperature on the structure and morphology of *O. tenuiflorum*-mediated ZnO NPs. The ZnO NPs were calcined at 300, 400, and 500 °C and the best quality NPs were obtained at 400 °C. The average diameter reduced from 36 to 30 nm with an increase in calcination temperature from 300 to 400 °C and an increase in calcination temperature after 400 °C resulted in an increase in particle size. The ZnO NPs were all spherical, however at 300°C the ZnO NPs were clustered, and the quality improved as calcination temperature was increased to 400 °C. Increasing the calcination temperature to 500 °C resulted in highly agglomerated large particles. However, Mfon and associates [101] observed an increase in the crystallite size with an increase in calcination temperature and plant concentration for *O. gratissimum*-mediated ZnO NPs. Moreover, the bandgap was reduced with an increase in calcination temperature and plant extract concentration.

From the observations above, reaction conditions can affect the morphology, size, and optical properties of ZnO NPs and controlling these parameters during synthesis can tune the properties of ZnO NPs for their intended applications. Therefore, there is a need to optimise the reaction conditions in order to obtain the best properties for the intended applications of the ZnO NPs. Chegini et al. [102] optimised plant extract concentration (10–50%) and pH (7–11) for the synthesis of ZnO NPs using *Satureja sahendica* plant extract. The optimum plant concentration and pH were found to be 40% and 10 respectively. Sushma and co-workers [89] also optimised the pH, reaction time, plant concentration, and Zn precursor concentration for *O. tenuiflorum*-mediated ZnO NPs synthesis. The *O. tenuiflorum*-mediated ZnO NPs were synthesised using 4–24 mM zinc acetate dihydrate, 125–750 µg *O. tenuiflorum* at pH 8–12, and for 30 min to 24 h. No surface plasmon resonance (SPR) peaks of ZnO NPs were observed from the UV-Vis at 16 nm and 24 mM Zn(CH_3_COO)_2_·2H_2_O and the optimum Zn salt precursor was 20 mM. All plant *O. tenuiflorum* concentrations exhibited SPR peaks of ZnO NPs from 350 to 380 nm and 250 µg was the optimum as it had a higher absorption intensity. The optimum pH was observed at 12. The reaction rate increased with reaction time and a 70% reduction was observed in just 30 min. Asjadi and Yaghoobi [103] investigated the effect of reaction parameters on synthesised *Mentha pepperita*-mediated ZnO NPs using a hydrothermal method. The formation of ZnO NPs were observed at 50 mM Zn(NO_3_)_2_·6H_2_O whereas no ZnO NPs was formed at 5 mM Zn(NO_3_)_2_·6H_2_O. The hydrothermal synthesis of *M. pepperita*-mediated ZnO NPs at a pH of 8 was proposed to be a dissolution-precipitation. This is because the self-assembled structure was formed in 8 h followed by shrinkage and disappearance of the structure in 16 h. Finally, after 24 h a smaller structure was formed again. The *M. pepperita*-mediated ZnO NPs synthesised at pH 8 and 12 for 24 h did not differ in morphology however they differed in particle size and crystallite size with those synthesised at pH 8 smaller in size.

**Table 1:** Different morphologies of Lamiaceae-mediated synthesis, particle size and the precursor used.

Type of Plant	Zn Precursor	Morphology	Particle Size	Ref
*Perilla frutescens*	Zinc nitrate	Triangular	-	[78]
*Mentha arvensis*	Zinc acetate dihydrate	-	30–100 nm	[81]
*Scutellaria baicalensis*	Zinc acetate dihydrate	Spherical	25–30 nm	[82]
*Plectranthus amboinicus*	Zinc nitrate hexahydrate	Rods	88 nm average size	[83]
*Ocimum basilicum*	Zinc acetate dihydrate	Non spherical	40 nm average size	[84]
*Mentha spicata*	Zinc acetate dihydrate	Nanorods	80–100 nm average size	[85]
*Anisomeles malabarica*	Zinc nitrate Zinc acetate	Round gathers into flowers Spherical gathers into bullets	1.5–8.5 nm average size	[87]
*Ocimum tenuiflorum*	Zinc acetate dihydrate	Rods	38–163 nm	[89]
*Isodon rugosus*	Zinc acetate dihydrate	Triangular	-	[90]
*Ocimum gratissimum*	Zinc acetate dihydrate	Spherical	38–68 nm	[91]
*Ocimum tenuiflorum*	Zinc nitrate hexahydrate	Nanorods	30 nm average diameter	[92]
*Tetradenia riperia*	Zinc nitrate hexahydrate	Spherical	64 nm average size	[94]
*Ocimum gratissimum*	Zinc chloride	Nanorods	54–87 nm diameter	[95]
*Mentha spicata*	Zinc nitrate hexahydrate	Scales and crystals	20–70 nm	[97]
*Anisochilus carnosus*	Zinc nitrate hexahydrate	Quasi-spherical	20–40 nm in diameter	[98]
*Ocimum gratissimum*	Zinc nitrate hexahydrate	Spherical	14–17 nm	[101]
*Satureja sahendica*	Zinc chloride	Multidimensional round	48–61 nm	[102]
*Ocimum americanum*	Zinc nitrate hexahydrate	Spherical	21 nm average size	[104]
*Ocimum basilicum*	Zinc nitrate hexahydrate	Hexagonal	<50 nm	[105]
*Betonica officinalis*	Zinc nitrate	-	10 nm	[106]
*Hyptis suaveolens*	Zinc nitrate	Hexagonal	10–200 nm	[107]
*Lavandula angustifolia*	Zinc acetate dihydrate	Aggregates with truncated and triangular	61.52 nm average size	[108]
*Leucas aspera*	Zinc acetate dihydrate	Spherical with a few rods	35.10 nm average crystallite size	[109]
*Mentha arvensis*	Zinc nitrate hexahydrate	Irregular	20–15 nm crystallite size	[110]
*Mentha pulegium*	Zinc nitrate hexahydrate	Quasi-spherical	40 nm average size	[111]
*Mentha pulegium*	Zinc nitrate hexahydrate	Spherical	65.02 nm average diameter	[112]
*Ocimum americanum*	Zinc acetate dihydrate	Spherical	50 nm	[113]
*Ocimum basilicum*	Zinc nitrate hexahydrate	Almost spherical	27 nm average size	[114]
*Rosmarinus officinalis*	Zinc nitrate hexahydrate	Aggregates of elongated shapes	-	[114]
*Ocimum basilicum*	Zinc acetate dihydrate	Irregular	10–25 nm	[115]
*Ocimum basilicum*	Zinc acetate dihydrate	Spherical	31 nm average size	[116]
*Ocimum basilicum*	Zinc acetate dihydrate	-	30–40 nm	[117]
*Ocimum tenuiflorum*	Zinc acetate dihydrate	Hexagonal	42 nm crystallite size	[118]
*Ocimum tenuiflorum*	Zinc nitrate hexahydrate	Hexagonal	11–25 nm diameter range	[119]
*Ocimum tenuiflorum*	Zinc hexahydrate	Spherical	10–20 nm diameter	[120]
*Ocimum tenuiflorum*	Zinc acetate dihydrate	Spherical	58.5 nm average diameter	[121]
*Ocimum tenuiflorum*	Zinc nitrate hexahydrate	Flakes	30–40 nm average size	[122]
*Origanum majorana*	Zinc sulphate heptahydrate	Rods	90–125 nm width	[123]
*Origanum vulgare*	Zinc nitrate hexahydrate	Spherical	20–30 nm	[124]
*Phlomis*	Zinc nitrate hexahydrate	Hexagonal	79 nm average size	[125]
*Plectranthus babatus*	Zinc acetate dihydrate	Spherical	30–60 nm	[126]
*Salvia officinalis*	Zinc acetate dihydrate	-	26.14 nm average size	[127]
*Scutellaria baicalensis*	Zinc nitrate hexahydrate	Spherical	50 nm	[128]
*Scutellaria baicalensis*	Zinc acetate dihydrate	Spherical	33.14–99.03 nm	[129]
*Solenostemon monostachyus*	Zinc nitrate	Spherical	23.06 nm average crystallite size	[130]
*Tectona grandis*	Zinc nitrate hexahydrate	Almost spherical	54 nm	[131]
*Tectona grandis*	Zinc nitrate hexahydrate	Spherical and agglomerated	124.6 nm	[132]
*Thymus spicata*	Zinc acetate dihydrate	Irregular and almost spherical	6.5–7.5 nm	[133]
*Thymus vulgaris*	Zinc nitrite	Spherical	46.74 nm crystallite size	[134]
*Thymus vulgaris*	Zinc acetate dihydrate	Popcorn-like	50 nm average size	[135]
*Vitex negundo*	Zinc nitrate hexahydrate	Spherical	75–80 nm	[136]
*Vitex trifolia*	Zinc nitrate hexahydrate	Spherical	28 nm average size	[137]

### 3.4. Mechanism of Formation of Lamiaceae-Mediated ZnO NPs

**Figure 2:**
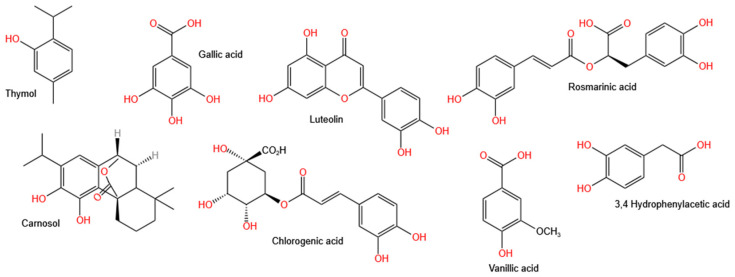
Polyphenolic compounds found in some Lamiaceae plants.

There have been very few reports on the mechanism of the formation of ZnO NPs prepared using plants belonging to the Lamiaceae family. This is not surprising given that the mechanism of the formation of ZnO NPs via plant-assisted synthesis is not well understood. This is owing to the possible involvement of several phytochemical compounds from the plant extracts in the synthesis of the ZnO NPs, which also vary in composition and concentration depending on the type of plant or plant part used. The Lamiaceae family is well-known for its high polyphenols content [138]. These polyphenols have been attributed to the high antioxidant properties of the Lamiaceae family [139], making them suitable candidates for the reduction of Zn salt precursors during ZnO NPs synthesis. Figure 2 shows some of the polyphenolic compounds found in Lamiaceae plant extracts [139]. The mechanism of formation of Lamiaceae-mediated ZnO NPs involves the formation of the Zn^2+^ complex with the aromatic phytochemical compounds via the hydroxyl functional groups. This is followed by the thermal decomposition and growth of the NPs resulting in capped and stabilised ZnO NPs.

**Figure 3:**
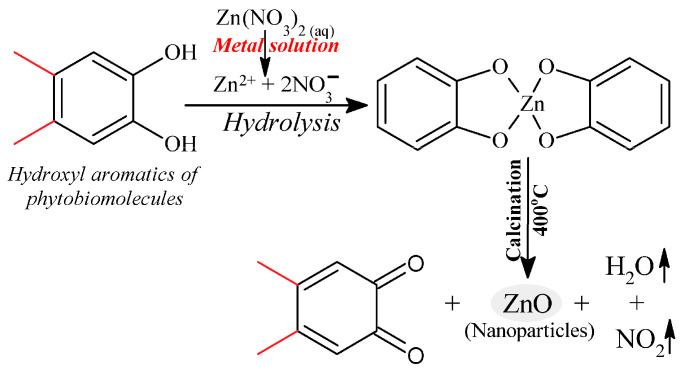
Mechanism of formation of *O. vulgare*-mediated ZnO NPs. Adapted from [124].

Kamli et al. [124] proposed a mechanism for the formation of ZnO NPs, which is shown in Figure 3. The mechanism involved quercetin, which is one of the major polyphenol aromatic hydroxyl biomolecules found in *O. vulgare* leaf extract forming a complex with Zn^2+^ ions from the Zn(NO_3_)_2_·6H_2_O. This was then followed by thermal composition via calcination at 400 °C to obtain the ZnO NPs.

A similar mechanism of ZnO NPs formation was proposed for the *Vitex negundo*-mediated ZnO NPs. The mechanism involved the formation of a complex between Zn^2+^ ions from the Zn(NO_3_)_2_·6H_2_O precursor and the isoorientin and luteolin, which are aromatic biomolecules with hydroxyl function groups to form a complex under acidic conditions. The ZnO NPs were obtained via the thermal decomposition of the complex [136]. Alamdari et al. [140] attributed the capping and stabilisation of *M. pulegium*-mediated ZnO NPs to the functional groups –C–C–, –C–O–, –C=C–, and –C=O from phytochemicals such as alkaloids, flavonoids, phenols, and anthracenes, which were observed from the Fourier transform infrared spectroscopy (FTIR) spectrum of *M. pulegium*-mediated ZnO NPs. Additionally, the authors also reported the disappearance of –OH functional groups observed from the FTIR spectrum of *M. pulegium* leaf extracts and attributed this to the –OH groups taking part in the reduction during the synthesis of ZnO NPs.

In another study using *R. officinalis* leaf extract to the synthesis ZnO NPs, the phenolic compounds in the *R. officinalis* leaf extract were proposed to be responsible for the stabilisation of the ZnO NPs. The reactions involved the dissolving the Zn(NO_3_)_2_·6H_2_O precursor in water to obtain Zn^2+^ ions. This was then followed by precipitation of the Zn^2+^ using NaOH and finally calcination leading to the thermal decomposition of the zinc hydroxide to ZnO NPs, as shown in the Equations below [93].

$$\mathrm{Zn}({\mathrm{NO}}_{3}{)}_{2}\cdot 6H_{2}O+\overset{H_{2}O}{\to }\;2\mathrm{Zn}2^{+}+2{{\mathrm{NO}}_{3}}^{-}+6H_{2}O$$

$${\mathrm{Zn}}^{2+}+2{\mathrm{OH}}^{-}\to \mathrm{Zn}(\mathrm{OH}{)}_{2}$$

$$\mathrm{Zn}(\mathrm{OH}{)}_{2}\;\overset{\Delta }{\to }\;\mathrm{ZnO}+H_{2}$$

More studies are necessary to better understand the mechanism of plant-mediated synthesis of ZnO NPs, which will lead to improved control and tuning of parameters during synthesis in order to yield high-quality ZnO NPs.

## 4. Characterisation Techniques Used for Lamiaceae-Mediated ZnO NPs

The characterisation of ZnO NPs after synthesis is necessary to confirm the formation of NPs, optimise the reaction conditions, and to investigate the properties of the NPs. The commonly used characterisation techniques for ZnO NPs include XRD, TEM, SEM, energy dispersive X-ray analysis (EDX), FTIR, and UV-Vis for structural, morphological, and optical properties and these are discussed below.

### 4.1. XRD

XRD is a prominent analytical technique that is non-destructive and is used to investigate chemical compounds. Information about crystal structure, phase, crystallinity, texture, and average crystallite size can be obtained from XRD analysis. The XRD principle involves scattering X-rays by atoms of a crystal leading to the formation of diffraction patterns and interferences of the X-rays with each other. The interferences can be detected using Braggs law, thus giving the crystal material characteristics. The average size of the crystal material can be estimated using the Scherer equation [141,142].

D = 0.94λ/β cos θ

where D is the crystalline size, λ is the X-ray wavelength, β is the full-width half maximum of the peak.

Lamiaceae-mediated ZnO NPs were all characterised using XRD. Zheng et al. [83] used XRD to investigate the nature and purity of *P. amboinicus*-mediated ZnO NPs. The authors reported that the synthesised ZnO NPs were pure and crystalline. XRD was used to determine the average crystal size of *O. tenuiflorum*-mediated ZnO NPs and the average crystallite size was calculated to be 13.86 nm [119]. Rani and co-workers [143] investigated the structure of *Hyssopus officinalis*-mediated ZnO NPs. The XRD results revealed a hexagonal wurtzite ZnO NPs structure, which is the structure of ZnO that is stable under RT and pressure. However, small peaks corresponding to zinc sulphate from the unreacted Zn salt precursor were also observed.

### 4.2. UV-Vis

UV-Vis is a low-cost analytical tool that is commonly used to confirm formation of NPs. The method involves the scanning of the ZnO NPs sample in the UV region from about 200 to 800 nm of the electromagnetic wave to obtain SPR bands [144]. The SPR band is produced due to the interactions between the oscillating surface electrons of the ZnO NPs and light. The SPR peaks of ZnO NPs have been reported to range from 289 to 385 nm wavelength, and it can be affected by morphology and particle size [145,146]. The UV-Vis analysis of *Hyptis suaveolens*-mediated ZnO NPs and an SPR peak was obtained at 375 nm, which indicated the formation of ZnO NPs [107]. This is similar to the SPR peak observed for *Vitex trifolia*-mediated ZnO NPs [137].

### 4.3. FTIR

FTIR is based on the absorption of infrared (IR) radiation by a molecule, modification of the dipole, and the molecule becoming IR active, resulting in a spectrum [147]. It is an important technique that is used to confirm the biomolecules from the plant extracts that are taking part in the synthesis of ZnO NPs. It can also be used to investigate the functional groups that are capped on the surface of the NPs. Additionally, the ZnO bond can also be confirmed using FTIR. Rad et al. [111] used FITR to investigate the biomolecules involved in the synthesis of ZnO NPs using *M. pulegium* leaf extract. The *M. pulegium* leaf extract exhibited absorption bands at 3440 and 2890 cm^−1^, which were assigned to amines stretching vibrations, alcohol O-H stretching, and alkane C-H stretching, whereas the absorption bands exhibited at 1725 and 1180 cm^−1^, which were assigned to aromatic C=C stretch and C-N stretching, respectively. The synthesised ZnO NPs exhibited absorption bands at 1591, 881, and 1382 cm^−1^, which were attributed to aromatic groups and their functional groups. These aromatic groups could be responsible for capping the ZnO NPs, thus preventing the agglomeration of the NPs. Additionally, the characteristic absorption of ZnO was observed at 495 cm^−1^, which confirms the synthesis of ZnO NPs. Absorption bands between 1362 and 872 cm^−1^ were observed from the FTIR spectra of *M. longifolia*-mediated ZnO NPs. The bands were assigned to the amine group from proteins or enzymes and phenol groups, respectively. These functional groups from the plant extract could be responsible for the stabilisation of the synthesised NPs [148].

### 4.4. SEM

SEM is an electron microscopy technique that is frequently used in the characterisation of NPs. It is used to determine the surface morphology, shape, size, and size distribution of NPs. It involves the bombardment of the surface of NPs with a high-energy electron beam and the formation of an image using the back-scattered electrons. SEM can provide information on the purity and aggregation; however, it does not provide the internal structure [149]. SEM can be coupled with EDX, which is used for chemical composition analysis.

### 4.5. EDX

EDX is an analytical technique that is used for analysing the chemical composition and purity of NPs. It is based on measuring the X-rays emitted by the NPs during bombardment with a high-energy electron beam. The X-rays are generated according to the characteristics of the elements present; thus, each element has its own unique atomic structure that generates distinct peaks. The measurement is given as the relative abundance of X-rays against their energy [142,150]. The EDX spectrum obtained from *P. amboinicus*-mediated ZnO NPs confirmed the presence of 75.12% zinc, 23.55% oxygen, and a small peak, which was attributed to the bound bio-compounds from the *P. amboinicus* extract [151]. In another study, the EDX spectrum of *S. baicalensis*-mediated ZnO NPs confirmed the presence of zinc and oxygen with a percentage of the weight as 34.58% and 63.5%, respectively. A tiny peak of 2.08% weight was also observed, and it was attributed to the biomolecules from *S. baicalensis* extract linked to the synthesised ZnO NPs [129].

### 4.6. TEM

**Figure 4:**
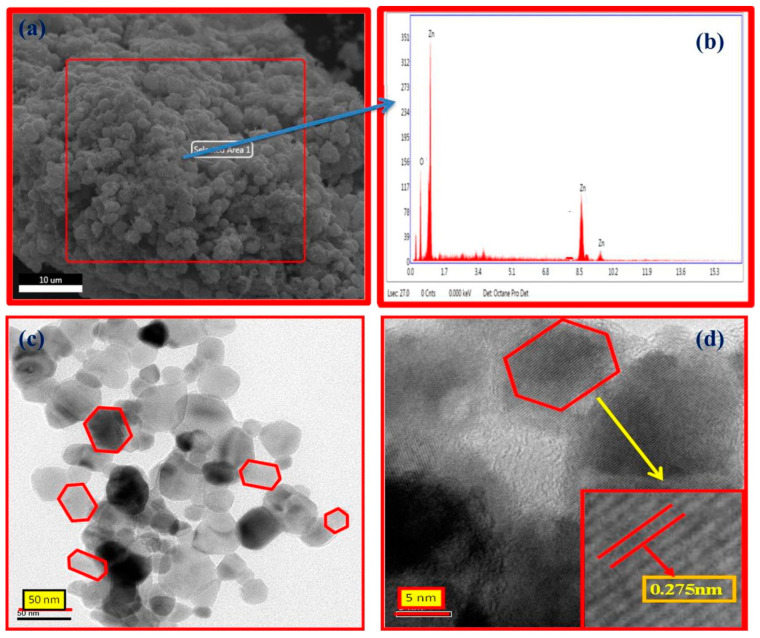
(**a**) SEM image (**b**) EDX (**c**) TEM, and (**d**) HR TEM images of *M. arvensis*-mediated ZnO NPs. Adapted from [110].

TEM is another powerful electron microscopy technique that is frequently used in the characterisation of NPs. It is used to determine the particle size, size distribution, and morphology of NPs. It involves the interaction of an electron beam with NPs and the formation of an image on a photographic plate. Additional information, such as the arrangement of atoms and their local structure can be obtained from a high-resolution transmission electron microscope (HRTEM), which is a high-resolution TEM [152]. Figure 4 shows the SEM, EDX, TEM, and HRTEM of *M. arvensis*-mediated ZnO NPs. The SEM image shows the agglomerated and irregularly shaped ZnO NPs whereas the TEM and HRTEM images show the nanoregime with hexagonal patterns and d spacings [110].

### 4.7. DLS

Dynamic light scattering (DLS) is a widely used technique to determine the particle size and distribution of particles over a range of sizes. The technique involves the measurement of light interferences as a function of time based on the Brownian motion of particles dispersed in the colloidal suspension and correlation to its velocity with the size estimated using the Stokes–Einstein equation. Additionally, size distribution is reported as a heterogeneous or homogeneous population using the polydispersity index (PDI) [153].

### 4.8. Zeta Potential

Zeta potential is a technique used to measure the surface charge of NPs in a colloidal solution. NPs have a thin layer of counter ions due to their surface charge and the Zeta potential is a measure of the electric potential at the boundary of this double layer. The Zeta potential is influenced by the pH of the medium, surface chemistry, particle concentration, temperature, size of the particle, solvent, and ionic strength [154]. The magnitude of the Zeta potential is indicative of the stability of the NPs in a solution, and generally, a Zeta potential of ±25–40 mV is considered to be stable, while greater than ±60 mV is considered highly stable [155]. Senthilkumar et al. [131] reported a Zeta potential of −25 mV for *T. grandis*-mediated ZnO NPs. The value of 25 indicated that the synthesised ZnO NPs were stable. Additionally, the researchers attributed the negative value to the reducing and stabilising agents, such as phenolic compounds from the *T. grandis* extract.

Additional characterisation techniques of the plant-mediated ZnO NPs include a thermogravimetric analyser (TGA) for thermal stability, Brunauer–Emmett–Teller (BET) for surface analysis, and X-ray photoelectron spectroscopy (XPS) for elemental composition. In addition to using FTIR, utilising chromatography tools such as gas chromatography equipped with mass spectrometry (GC-MS) and high-performance liquid chromatography equipped with mass spectrometry (HPLC-MS), and liquid chromatography equipped with mass spectrometry (LC-MS) can aid with the analysis of plant extracts before and after synthesis. This can help in deducing some of the biomolecules that could be involved in the synthesis.

## 5. Antibacterial Activity of ZnO NPs

Antibacterial activity is the action of destroying bacteria or inhibiting of bacterial growth with antibacterial agents, which destroy or inhibit bacteria growth. Bacteria are characterised by a cell wall, cell membrane, and cytoplasm. They can be classified into Gram-positive (G+) and Gram-negative (G−) strains, whereby G+ strains have a thick peptidoglycan layer of around 20–80 nm in the cell wall and G− strains have a thin peptidoglycan layer of around 7–8 nm in the cell walls [156]. Antibiotic resistance has been on the rise in recent years, posing a severe threat to life [157]. Therefore, safe, cheaper, and better antibacterial agents are needed to combat the threat of antibiotic resistance. Plant-mediated ZnO NPs are safe, cheaper, and have shown superior antibacterial activity compared to chemically synthesised ZnO NPs, making them a promising solution to overcome antibiotic resistances.

Different analytical methods, such as broth dilution, agar dilution, disk diffusion, and microtiter plate-based methods are utilised to investigate the antibacterial activity in vitro. The broth dilution method is the most common analytical method to assess antibacterial activity, followed by colon count [156]. The antibacterial assessment of ZnO NPs is given as the measure of the zone of inhibition (ZOI), which is an area where there is no growth of bacteria when the NPs are applied suspended in dimethyl sulfoxide (DSMO).

### 5.1. Mechanism of Antibacterial Activity of ZnO NPs

**Figure 5:**
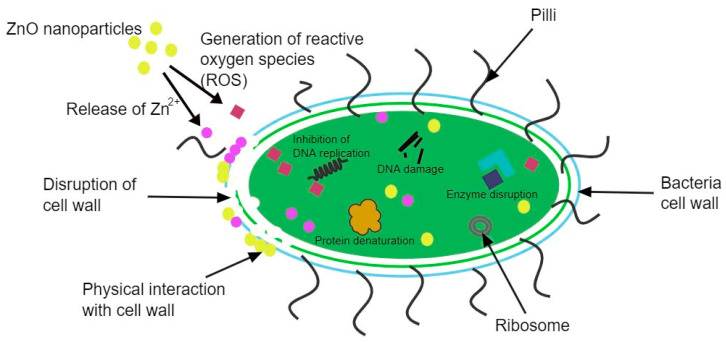
Mechanisms of antibacterial activity of ZnO NPs.

There are several mechanisms of antibacterial activity of ZnO NPs that have been proposed, as shown in Figure 5, and these can be classified into physical and chemical interactions between ZnO NPs and the bacteria. The commonly accepted chemical mechanism involves the production of reactive oxygen species (ROS), such as superoxide and hydrogen peroxide. The produced ROS damages the cells and the components in the cell through oxidative stress leading to the disfunction, and eventually, the death of the bacteria [158].

This process can be illustrated by the equations below, and it is similar to the photocatalysis mechanism.

$$h^{+}+H_{2}O\to \cdot \mathrm{OH}+H^{+}$$

$$e^{-}+O_{2}\to \cdot O_{2}{}^{-}$$

$${O_{2}}^{-}+H^{+}\to HO_{2}$$

$${\mathrm{HO}}_{2}\cdot +H^{+}+e^{-}\to H_{2}O_{2}$$

The process begins when the ZnO NPs are radiated with UV light, resulting in the excitation of electrons from the valence band (VB) to the conduction band (CB), thus creating electrons (e^−^) and holes (h^+^). The h^+^ splits the water in an oxidate process resulting in **·**OH, whereas the e^−^ combines with the dissolved oxygen in a reduction process, generating **·**O_2_^−^. This is then followed by the superoxide radical reacting with the H^+^ yielding HO_2_**·**, which then collides with the e^−^, resulting in a hydrogen peroxide radical. Finally, the hydrogen peroxide radical reacts with the H^+^ generating H_2_O_2_. Due to the negative charge of **·**OH and **·**O_2_^−^, cannot penetrate the cell. However, the generated hydrogen peroxide is able to penetrate the cell and destroy the bacteria. This mechanism of antibacterial activity is largely dependent on the size of the ZnO NPs. The smaller the NPs, the larger the surface area, and the higher the hydrogen peroxide generation resulting in higher antibacterial activity [159].

The second proposed mechanism of ZnO NPs antibacterial activity involves the release of Zn^2+^. The mechanism is still not fully understood; however, it has been proposed to be based on the toxicity of ZnO, which is often attributed to the release of Zn^2+^ ions. The Zn^2+^ ions can be adsorbed on the surface of the bacteria where they disrupt the cell membrane and penetrate the intercellular components. While inside the cell, the Zn^2+^ ions interact with the organic functional groups, resulting in an unbalanced metabolism, thus leading to the destruction of the bacteria [160].

Another mechanism that has been proposed is based on the physical interactions between the ZnO NPs and the bacteria surface. The interactions can be Van der Waals forces, electrostatic attractions, hydrophobic interactions, and receptor-ligand interactions. These interactions change the charge balance of the bacteria’s surface, resulting in the deformation of the cell and the destruction of the bacteria. ZnO NPs with a positive Zeta potential are adsorbed on the surface of negatively charged bacteria via electrostatic attractions, whereas ZnO NPs with a negative Zeta potential are adsorbed via receptor-ligand interactions [161].

The antibacterial mechanism of ZnO NPs has been studied, and the exact mechanism is still not fully understood, hence additional studies are needed to fully understand the mechanism before ZnO NPs can be employed as antibacterial agents commercially.

### 5.2. Antibacterial Activity of Lamiaceae-Mediated ZnO NPs

The antibacterial activity of ZnO NPs is dependent on the synthesis method of the NPs, the type of plant extract used for the synthesis and the type of bacteria, and the minimum inhibitory concentration (MIC), which is the minimum concentration of the NPs in a suspension that is required to effect visible bacteria inhibition. Rather et al. [108] investigated the antibacterial activity of *Lavandula angustifolia*-mediated ZnO NPs against G+ *Straphylococcus aureus* (*S. aureus*) and G− *Escherichia coli *(*E. coli*) and studied the effect of different concentrations (25, 50, 70, and 100 µg/mL) of *L. angustifolia*-mediated ZnO NPs against the bacteria. The ZOI for 25, 50, 70, and 100 µg/mL *L. angustifolia*-mediated ZnO NPs were 12.66, 15.33, and 20.33 mm, and 12.33, 14.66, and 15.0 mm for *S. aureus* and *E. coli,* respectively. The ZOI increased with an increase in ZnO NPs concentration and with the *L. angustifolia*-mediated ZnO NPs exhibiting good antibacterial activity against both G+ and G− bacteria. However, the maximum ZOI was higher against the G+ bacteria. According to Vijayakumar et al. [126], this is due to G− having an outer lipopolysaccharide membrane surrounding their thin peptidoglycan cell wall making it difficult for NPs to penetrate the cells. Saini and associates [123] also observed a greater antibacterial activity of *Origanum majorana*-mediated ZnO NPs against the G+ *S. aureus* and *Streptococcus pneumonia* (*S. pneumonia*) bacteria compared to the G− *Pseudomonas aeruginosa* (*P. aeruginosa)* and the *E. coli* bacteria and using the broth diffusion method. The MICs were reported to be 100–125 µg/mL for *E. coli*, 150–175 µg/mL for *P. aeruginosa,* and 76–100 µg/mL for both *S. aureus* and *S. pneumonia.* However, this differs from the findings by Alyamani et al. [125] using *Phlomis*-mediated ZnO NPs, who reported a higher maximum ZOI against G− *E. coli* (16.8 mm) compared to the G+ *S. aureus* (15.1 mm) at 2000 µg/L *Phlomis*-mediated ZnO NPs. This was attributed to the thicker peptidoglycan layer of G+ bacteria compared to G− bacteria, making it more difficult for NPs to penetrate. However, when the mechanism of antibacterial activity involving ROS is considered, the differences in antibacterial activity for G+ and G− bacteria may be due to different intercellular reactions. This might explain the disparities in the data on the sensitivity of G+ and G− bacteria to ZnO NPs.

The synthesis method of ZnO NPs can have an effect on the antibacterial activity of ZnO NPs. This was demonstrated by Kurian and Elumalai [109], who carried out comparative antibacterial activity studies using L. aspera-mediated ZnO NPs and chemically mediated ZnO NPs against G− Aeromonas hydrophila (A. hydrophila) and G+ Streptococcus agalactiae (S. agalactiae) using the disc diffusion method. The plant-mediated ZnO NPs exhibited better antibacterial activity against both the G+ and the G− bacteria compared to the chemically derived ZnO NPs. In another study, Stan et al. [114] investigated the antibacterial activity of chemically derived, R. officinalis-mediated, O. basilicum-mediated ZnO NPs, and ZnO NPs synthesised using Allium sativum belonging to the Liliaceae family against different bacteria. A. sativum-mediated ZnO NPs exhibited the highest antibacterial activity against all the bacteria. Despite the fact that R. officinalis-meditated ZnO NPs had larger particles (54 nm) compared to the chemically synthesised ZnO NPs (25 nm), they still had a better antibacterial activity, as demonstrated by the ZOI of 19.2 mm and MIC of 0.39 µg/mL compared to the chemically derived ZnO NPs with ZOI of 16.3 mm and MIC of 12.5 µg/mL. Plant-mediated has been reported to exhibit better antibacterial activity compared to chemically synthesised ZnO NPs. This can be attributed to the presence of bioactive compounds from the plant extracts that could be capped on the surface of the ZnO NPs to stabilise the NPs.

Irshad and associates [117] reported better antibacterial activity for the O. basilicum-mediated ZnO NPs compared to the Gentamycin antibiotic control. The ZnO NPs were active against S. aureus and E coli with a ZOI of 31.05 and 36.15 mm, respectively, and with a MIC of 312.5 µg/mL for both S. aureus and E. coli, compared to 25 and 26 mm for Gentamycin, respectively. This is in agreement with the observations by Vidhya and associates [113] for O. americanum-mediated ZnO NPs against G+ (*Bacillus cereus* (*B. cereus*), *Clostridium perfringens* (*C. perfringens*)) G− (*Klebsiella pneumoniae* (*K. pneumoniae*), *Salmonella Paratyphi* (*S. paratyphi*)). The *O. americanum*-mediated ZnO NPs exhibited a greater antibacterial activity against both the G+ and the G− bacteria compared to the Gentamycin antibiotic control.

The particle size of ZnO NPs is known to influence antibacterial activity. Smaller particles result in an enhanced antibacterial activity owing to an increased surface area for the generation of ROS or interaction with the bacteria cell wall [162]. Moreover, the smaller the particle size, the easier it is for the particles to penetrate the bacteria membrane. This was demonstrated by Zare et al. [134] when they utilised different T. vulgaris leaf extract concentrations to synthesise ZnO NPs, resulting in different particle sizes of ZnO NPs. The 1 mL plant extract had smaller-sized particles, while the 0.5 mL plant extract resulted in larger particles with aggregation. The synthesised ZnO NPs were investigated for antibacterial activity, and it was observed that the antibacterial activity increased with a decrease in particle size. This was attributed to the larger specific surface area of smaller particles and the increased number of bioactive compounds capped on the surface of the ZnO NPs synthesised from the 1 mL plant extract (smaller particles).

The antibacterial of ZnO NPs can be enhanced by doping or composites. Green synthesised ZnO NPs and Ag-ZnO NPs synthesised *T. riperia* were evaluated for antibacterial activity and the Ag-ZnO NPs exhibited a better antibacterial activity, as compared to the ZnO NPs against both the G+ *S. aureus* and the G− *E. coli* bacteria. The antibacterial activity increased with an increase in Ag doping concentration showing that Ag improves the antibacterial activity of the ZnO NPs. The improved antibacterial activity was attributed to the Ag synergetic effect and also due to the smaller particles of Ag-ZnO NPs compared to ZnO NPs [94]. To reduce the amounts of NPs that organs and tissues in the body may be exposed to and accumulate when using NPs based antibacterial, the NPs can be mixed with bacteriophages (phages). This was demonstrated for *O. basilicum*-mediated ZnO by Abdelsattar et al. [115]. The authors reported a better antibacterial activity of 0.6 mg/mL ZNO NPs and a phage ZCSE6 at Multiplicity of infection (MOI) of 0.1 compared to 0.6 mg/mL ZnO NPs against *Salmonella enterica*. The improved antibacterial activity can be attributed to the synergic effect of the ZnO NPs and phage mixture.

The Lamiaceae-mediated ZnO NPs have exhibited a greater antibacterial efficacy compared to the chemically derived ZnO NPs, and, in some cases, standard antibiotics. It has been demonstrated that factors such as concentration of ZnO NPs, particle size, type of bacteria, and synthesis methods affect the antibacterial activity of the ZnO NPs. Moreover, the composites of ZnO NPs and other antibacterial materials, as well as doping with other metals can improve the antibacterial activity of ZnO NPs. Table 2 shows the antibacterial activity of some Lamiaceae-mediated ZnO NPs, including the concentration of the ZnO suspension, the method of antibacterial assessment and the type of bacteria inhibited. From the table, the Lamiaceae-mediated ZnO NPs have shown antibacterial inhibition against different kinds of bacteria, making them possible candidates for antibacterial agents against antibiotic-resistant bacteria. However, additional studies on the effect of other parameters, such as incubation time, light availability, and morphology, are needed, as well as a better understanding of the mechanism of antibacterial activity.

## 6. Photodegradation Activity of Lamiaceae-Mediated ZnO NPs

### 6.1. Organic Dyes

**Figure 6:**
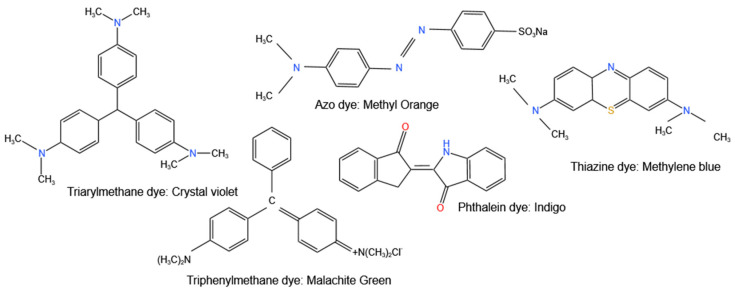
Classification of some organic dyes using their chromophores.

A dye is an organic molecule that has an affinity to a substrate to which it is being applied. Dyes are able to give colour because they absorb visible light and reflect a fraction of the light they do not absorb. They consist of two major constituents, which are chromophores and auxochromes. The chromophores are the functional groups, such as OH, SO_3_H, and NH_2_, which are responsible for imparting colour, whereas the auxochromes are the water-soluble parts, which are responsible for binding the dye to the substrate e.g., fibres. Classification of dyes is very complex because they can be classified based on the source of the dye, their applications, and functional groups [163,164]. Figure 6 shows classifications of some dyes using their chromophores.

### 6.2. Principle of Photodegradation Using ZnO NPs

**Figure 7:**
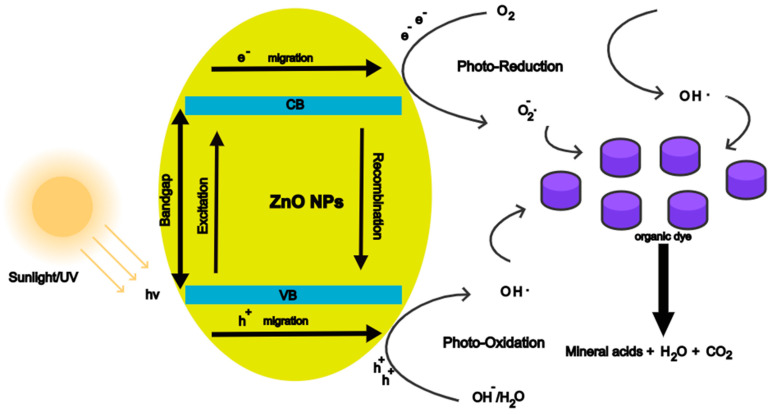
Mechanism of photodegradation of organic dye.

Photocatalysis is based on solar energy converting into chemical energy generating a catalytic reaction that will undergo reduction and oxidation reactions with oxygen and water, thus generating highly oxidising radicals [165]. When ZnO NPs are irradiated with energy (hv) that is equal to or greater than the bandgap, electrons (e^−^) are excited from the valence band (VB) to the conduction (CB), thus leaving holes in the VB. This creates electron-hole (e^−^/h^+^) pairs, as shown in Figure 7.

The electrons diffuse to the surface of the ZnO NPs and reduce the oxygen adsorbed on the surface to superoxide radical (O_2_^−^). The holes migrate to the surface and oxidise water-producing hydroxyl radicals **·**OH. The superoxide radicals react with H^+^ ions producing **·**HO_2_, H_2_O_2,_ and **·**OH. The hydroxyl radical is a powerful oxidising agent that can reduce the dye molecules adsorbed on the surface of the ZnO NPs to mineral acids, carbon dioxide, and water [166]. The equations for the process are given below.

$$\mathrm{ZnO}+\mathrm{hv}\to e^{-}{}_{\left(\mathrm{CB}\right)}+h^{+}{}_{\left(\mathrm{VB}\right)}$$

$$e^{-}+O_{2}\to \cdot {O_{2}}^{-}$$

$$h^{+}+H_{2}O\to \cdot \mathrm{OH}+H^{+}$$

$${O_{2}}^{-}+H^{+}\to \cdot {\mathrm{HO}}_{2}$$

$$2\cdot {\mathrm{HO}}_{2}\to H_{2}O_{2}+O_{2}$$

$$H_{2}O_{2}+e^{-}\;\to \cdot \mathrm{OH}+{\mathrm{OH}}^{-}$$

$$\mathrm{Dye}+\cdot \mathrm{OH}\;\to \text{Mineral acids}+{\mathrm{CO}}_{2}+H_{2}O$$

### 6.3. Photodegradation of Organic Dyes Using Lamiaceae-Mediated ZnO NPs

This section will discuss the photocatalytic activity of Lamiaceae-mediated ZnO NPs in degrading organic dyes as well as the effect of parameters such as photocatalyst dosage, reaction time, pH, particle size, initial dye concentration, and temperature, which are known to influence the photoactivity of ZnO NPs. Additionally, an improvement of the photoactivity of ZnO NPs by doping and coupling with other materials will be discussed.

Plant-mediated ZnO NPs have been used for the photodegradation of organic dyes such as methylene blue (MB) dye with excellent results. Chen et al. [128] reported 98.6% degradation of MB dye in 210 min under UV radiation using *S. baicalensis*-mediated ZnO NPs. Anbuvannan et al. [98] investigated the photodegradation of MB dye using *A. carnosus-*mediated ZnO NPs under UV irradiation. The intensity of the absorption peaks decreased with time, up to 90 min. The decrease in absorption peak intensity is an indicator of the photodegradation of MB dye. The high degradation efficiency was attributed to an increase in the surface defects, which lowered the recombination of the electron-hole pairs resulting in increased photoactivity. However, it should be noted that the presence of native defects in ZnO NPs can either reduce or increase the photoactivity and this depends on the type of defects and the location of these defects [167].

The photocatalytic efficiency of ZnO NP of organic dyes can also be influenced by the particle size and texture of the ZnO NPs. The smaller particles have a larger specific surface area compared to the larger particles. The larger specific area results in more active sites for photocatalysis to take place and better adsorption of UV light leading to an enhanced generation of ROS thus improved photoactivity [168]. However, an optimum size needs to be achieved because if the particles become too small, the recombination of the electron holes becomes prevalent, thus the photocatalysis will be reduced [169]. Algarni et al. [93] reported photocatalysis of *R. officinalis*-mediated ZnO NPs against MB dye and crystal violet (CV), the ZnO NPs synthesised at 80 °C exhibited the highest photocatalysis efficiency under sunlight irradiation at 40 °C with a photodegradation of 99.6% in 45 min for MB dye, and 99.9% for CV in 60 min. This was due to the smaller size, larger specific area, and higher pore volume of 14.7 nm, 12.0 m^2^/g, and 0.24 cm^3^/g, respectively, for the synthesised *R. officinalis*-mediated ZnO NPs at 80 °C compared to the 15.5 nm, 8.6 m^2^/g, and 0.12 cm^3^/g, respectively, synthesised at 180 °C.

Most studies on the photocatalysis of the activity of ZnO NPs against organic dyes report the negligible absorption of the organic dye molecules on the ZnO NPs. However, it has been reported that adsorption is crucial in the photodegradation of organic dyes, with high adsorption of dye molecules on the surface of the catalyst resulting in a higher photodegradation [170]. Asjadi and Yaghoobi [103] reported an adsorption efficiency of 49.3% and a photodegradation efficiency of 91.6% of MB dye using 0.05 g *M. pepperita*-mediated ZnO NPs under UV irradiation for 24 h. This was attributed to a decrease in agglomeration as a result of using high pressure during the hydrothermal synthesis of the *M. pepperita*-mediated ZnO NPs, and more surface modifications of the ZnO NPs by the biomolecules from the *M. pepperita* leaf extracts.

The photoactivity of *P. amboinicus*-mediated ZnO NPs, bare hydrothermally synthesised ZnO, and TiO_2_-P25 under UV irradiation for 180 min against Methyl red dye was investigated. The *P. amboinicus*-mediated ZnO NPs exhibited a better photodegradation efficiency of 92.45%, compared to 67.47% and 57.46% for bare ZnO and TiO_2_-P25, respectively. This enhanced photodegradation was attributed to the presence of long chains from biomolecules on the surface of the *P. amboinicus*-mediated ZnO NPs from the *P. amboinicus* leaf extracts. This is because phytochemicals, which function as capping agents for plant-mediated ZnO NPs, also assist in charge separation, and this reduces electron-hole pair recombination, leading to better photocatalysis [171].

The presence of biomolecule-capping agents from the plants was also attributed as the reason for the improved photodegradation of Malachite green using *Mentha arvensis-mediated* ZnO NPs reported by Stoyanova et al. [81]. A 74% discolouration was reported for *M. arvensis*-mediated ZnO NPs compared to 45% for bare ZnO NPs under UV irradiation for 120 min. However, the authors also attributed the enhanced photoactivity of the synthesised ZnO to the presence of the defects on the surface of the *M. arvensis*-mediated ZnO NPs, as well as a large specific surface area (80–140 m^2^/g) and a larger share of mesopores (pore sizes: 16–17 nm). This is because photocatalysis is complex in nature and it is influenced by more than one property. Particle size, surface defects, specific area, and morphology are some of the properties that influence the photoactivity of ZnO NPs.

Zare et al. [134] reported a better photodegradation efficiency of MB dye using *T. vulgaris*-mediated ZnO NPs, compared to TiO_2_ P-25. The improved efficiency was attributed to a greater mobility of the ZnO NPs electrons. The authors also investigated the effect of photocatalyst dosage on the reduction of MB dye using from 0.4 to 0.8 g/L *T. vulgaris*-mediated ZnO NPs. The reduction increased with an increase in dosage up to 0.6 g/L with a 96% reduction. This is due to the availability of more active sites for the reduction to take place. However, when ZnO NPs are in excess of agglomeration of particles and scattering of light occurs, hindering the reduction of the dye [172]. This was observed by Sharma et al. [92] for the photodegradation of 10 ppm Methyl orange dye (MO) using *O. tenuiflorum*-mediated ZnO NPs under UV irradiation for 180 min. The *O. tenuiflorum*-mediated ZnO nanorods exhibited a higher photodegradation efficiency and the degradation increased with an increase in ZnO nanorods dosage from 25 to 50 mg, and subsequently decreased at a 100 mg dosage. The photodegradation efficiency of *O. tenuiflorum*-mediated ZnO nanospheres, on the other hand, was about the same at between 25 and 50 mg but declined when the dosage was raised to 100 mg.

Luque et al. [96] investigated the photodegradation of Rhodamine B dye under UV and solar light irradiation using *O. vulgare*-mediated ZnO NPs. The degradation efficiencies were reported to be 94.4% for 100 min and 93.0% for 180 min for visible light and UV radiation, respectively. The enhanced photodegradation under solar light using *O. vulgare*-mediated ZnO NPs (bandgap −2.94 eV) was attributed to the presence of organic molecules on the synthesised ZnO NPs from the plant extract. These organic molecules can act as photosensitisers, allowing the ZnO NPs to absorb in the visible region of the spectrum.

The photocatalytic activity of ZnO NPs can be enhanced by doping with alkali metals, transition metals, rare earth metals, and non-metals. Additionally, coupling the ZnO NPs with carbon-based materials, such as graphene oxide (GO) is also known to improve the photocatalytic activity of ZnO NPs [173,174]. This is because doping with metals or non-metals reduces the bandgap of ZnO NPs, allowing the ZnO NPs to absorb in the visible region of the solar spectrum, and thus improve the photocatalytic activity. Carbonaceous materials serve as reservoirs for the photogenerated electrons, assist in the charge transfer of electrons, and as photosensitisers, which improves light absorption [161]. Patil et al. [175] investigated the photodegradation activity of *O. tenuiflorum*-mediated Ag-ZnO composite NPs. The reduction of the rhodamine B organic dye concentration increased by 5%, and the degradation time decreased by 3 h when compared to *O. tenuiflorum*-mediated ZnO NPs. The authors attributed the enhanced photocatalytic activity to the decreased recombination in Ag-ZnO NPs, the absorption of both UV and visible light of Ag-ZnO NPs, and silver assisting with the separation of charge.

In another study, the photodegradation of Rhodamine B dye using *O. basilicum*-mediated ZnO and RGO-ZnO NPs was investigated. RGO-ZnO NPs exhibited a higher photoreduction (96.7%) of the dye compared to ZnO NPs (91.4%) under visible light for 120 min. The increased photoreduction was attributed to RGO facilitating efficient electronic transmission channels, reducing charge recombination, and the synergic effect of RGO and ZnO, which promotes charge separation [116].

Most of the studies on the photodegradation of organic dyes using Lamiaceae-mediated ZnO NPs did carry out optimisation studies of the photocatalysis process. Reaction conditions, such as dosage, precursor concentration, reaction temperature, pH, etc., are known to affect the photodegradation efficiency, hence these parameters should be optimised in order to achieve the maximum photodegradation of the organic dyes. Abomuti and associates [127] reported the effect of reaction temperature, initial dye concentration, and catalyst dosage on the photodegradation of MO using *S. officinalis*-mediated ZnO NPs under UV irradiation. The photodegradation was time-dependent with a remarkable efficiency of 92.4% after 120 min. The influence of reaction temperature (300–330 K), initial dye concentration (5–20 ppm), and photocatalyst dosage (10–40 mg) were investigated. An increase in the MO dye concentration resulted in a decrease in absorbance, whereas the degradation rate increased with an increase in *S. officinalis*-mediated ZnO NPs concentration. An increase in the reaction temperature also led to an increase in the degradation rate.

Table 3 gives a summary of the percentage removal of the organic dyes using some Lamiaceae-mediated ZnO NPs. From Table 3, it can be seen that the photoactivity of Lamiaceae-mediated ZnO NPs is very remarkable. Nonetheless, these efficiencies are based on model dyes, and it would be interesting to note how these photocatalysts would perform when used for the photodegradation of real industrial wastewater.

## 7. Conclusions and Future Perspective

ZnO NPs can be successfully prepared using plant-mediated synthesis, which is a simple, cheap, and environmentally friendly approach. Plants belonging to the Lamiaceae family have been widely studied, and their interesting aromatic polyphenols can be used for the reduction of Zn salt precursors to ZnO NPs and stabilisation of the ZnO NPs. However, the mechanisation of formation is not yet fully understood, and future work can focus on investigating the mechanism of ZnO NPs formation using plant extracts. Synthesis conditions, such as pH, reaction temperature, calcination temperature, plant extract concentration, and Zn salt precursor concentration, had an influence on the size, morphology, and other properties of Lamiaceae-mediated synthesis. Therefore, controlling these parameters can assist in tailoring the properties of the NPs for a wide range of applications. Future work on the Lamiaceae can focus on the optimisation of the phytochemical extraction process and the ZnO NPs synthesis process.

This review highlighted the antibacterial activity and photodegradation of organic dyes using ZnO synthesised using plants belonging to the Lamiaceae family. The Lamiaceae-mediated ZnO NPs exhibited great antibacterial activity and photodegradation efficiency. They performed better than chemically synthesised ZnO NPs, thus demonstrating that plant-mediated synthesis is capable of producing ZnO NPs that can be employed in antibacterial and photocatalysis applications. The antibacterial activity can be influenced by the concentration of ZnO NPs, particle size, and type of bacteria, and changing these can result in great antibacterial activity. While photocatalysis is influenced by particle size, surface defects, and reaction parameters such as pH, reaction time, photocatalyst dosage, initial dye concentration, etc. However, for the photodegradation of organic dyes using Lamiaceae-mediated ZnO NPs, optimisation of these photodegradation reaction parameters was not well reported. Therefore, future work can look into the optimisation of the photodegradation reaction parameters for the effective photodegradation of organic dyes. Additionally, the antibacterial activity of ZnO NPs and photodegradation of organic dyes using ZnO NPs can be enhanced by doping or coupling with other metal oxides and carbonaceous materials. Additional studies can investigate the photodegradation of real industrial wastewater using the Lamiaceae-mediated ZnO NPs and the effect of incubation time, light availability, and morphology are needed, as well as a better understanding of the mechanism of antibacterial activity.

## Figures and Tables

**Table 2:** The ZOI, method, and type of inhibited bacteria for some Lamiaceae-mediated ZnO NPs.

Plant	Type of Bacteria	Method	Concentration of ZnO	Inhibition	Ref
*T. riperia*	*S. auerus* *E. coli*	Agar disk diffusion	1.5 mg/mL	7.67 mm 8.33 mm	[94]
*A. carnosus*	*S. paratyphi* *Vibrio cholerae* *S. aureus* *E. coli*	Disc diffusion	-	6 mm 10 mm 7 mm 9 mm	[98]
*Lavandula angustifolia*	*E. coli* *S. aureus*	Well diffusion assay	50 µg/mL	12.33 mm 12.66 mm	[108]
*O. americanum*	*Bacillus cereus* *Clostridium penfrigens* *Klebsiella Pnemoniae* *S. paratyphi*	Agar well	-	25 mm 30 mm 27 mm 24 mm	[113]
*O. basilicum*	*P. aeruginosa* *E. coli* *S. aureus* *Bacillus subtilis*	Disc diffusion	50 µL	10 mm 16 mm 14 mm 13 mm	[105]
*O. basilicum*	*S. aureus* *Salmonella triphimurium* *E. coli* *Listeria monocytogenes* *B. subtilis* *P. aeruginosa*	Agar diffusion	100 µg/mL	19.3 mm 8.2 mm 13.2 mm 11.4 mm 9.3 mm 12.4 mm	[114]
*R. officinalis*	*S. aureus* *Salmonella triphimurium* *E. coli* *Listeria monocytogenes* *B. subtilis* *P. aeruginosa*	Agar diffusion	100 µg/mL	19.2 mm 9.3 mm 12.7 mm 11.5 mm 9.0 mm	[114]
*O. basilicum*	*Salmonella enterica*	Disk diffusion Well diffusion	0.2 mg/mL	14.3 mm 12.3 mm	[115]
*Phlomis*	*E. coli* *S. aureus*	Disc diffusion	2000 µg/mL	16.8 nm 15.1 nm	[125]
*P. barbatus*	*B. subtilis* *Vibrio parahaemolyticus* *Proteus vulgaris*	Agar well diffusion	100 µg/mL	19.0 mm 15.0 mm 14.0 mm	[126]
*T. grandis*	*S. auerus* *B. subtilis* *E. coli* *S. paratyphi*		100 µg/mL	28 mm 30 mm 32 mm 29 mm	[131]

**Table 3:** The percentage removal of organic dyes using some Lamiaceae-mediated ZnO NPs.

Radiation Type	Plant	Pollutant	Concentration of Dye	% Removal	Time	Ref
UV light	*M. arvensis*	Malachite green	10 ppm	74%	120 min	[81]
Sun light	*R. officinalis*	MB	10 mg/L	99.64%	45 min	[93]
UV light Solar light	*O. vulgare*	Rhodamine B	15 ppm	94.24% 93%	100 min 180 min	[96]
UV light	*A. carnosus*	MB	10^−4^ M	-	90 min	[98]
UV light	*S. officinalis*	MO	5 ppm	92.47%	120 min	[127]
UV light	*S. baicalensis*	MB	50 µM	98.6%	210 min	[128]
UV light	*P. amboinicus*	Methyl red	10^−4^ M	92.45%	180 min	[151]
